# Factors Affecting the Global Health and Cultural Competencies of Nursing Students

**DOI:** 10.3390/ijerph19074109

**Published:** 2022-03-30

**Authors:** Mi-Kyoung Cho, Mi Young Kim

**Affiliations:** 1 Department of Nursing Science, Chungbuk National University, 1 Chungdae-ro, Seowon-gu, Cheongju 28644, Korea; ciamkcho@gmail.com; 2 College of Nursing, Hanyang University, 222 Wangsimni-ro, Seongdong-gu, Seoul 15588, Korea

**Keywords:** global health, cultural, competencies, nursing, students

## Abstract

Recently, various global health issues, including coronavirus disease 2019 (COVID-19), have been observed in relation to rapid changes in world health conditions; consequently, nurses’ global health and cultural knowledge have become increasingly important. Therefore, this study aimed to identify factors affecting the global health and cultural competencies of nursing students. The study design was a cross-sectional study with 108 participants; all participants were fourth-year nursing college students in S and C cities. Global health competency, cultural competency, global health confidence, cultural nursing confidence, and metacognition were surveyed online, and the data were collected from October 30 to November 7, 2018. The collected data were analyzed using descriptive statistics, independent t-test, Pearson’s correlation, and hierarchical multiple regression. The total mean scores for global health competency and the cultural nursing competency were 63.01 ± 8.78 and 134.94 ± 17.78, respectively. Global health competency had a positive correlation with cultural competency (r = 0.49, *p* < 0.001), cultural nursing confidence (r = 0.26, *p* = 0.006), and metacognition (r = 0.22, *p* = 0.023). Cultural competency showed a positive correlation with global health confidence (r = 0.31, *p* = 0.001), cultural nursing confidence (r = 0.51, *p* < 0.001), and metacognition (r = 0.40, *p* < 0.001). Cultural competency was found to be a significant factor affecting global health competency, with an explanatory power of 23.1% (F = 17.10, *p* < 0.001). Cultural nursing confidence and metacognition had significant effects on cultural competency, with an explanatory power of 34.3% (F = 14.97, *p* < 0.001). Cultural confidence and metacognition were important factors influencing cultural competency, and cultural competency was shown to be an important factor influencing global health competency.

## 1. Introduction

The coronavirus disease 2019 (COVID-19) pandemic poses a tremendous public health challenge worldwide. The pandemic has had global effects, leading to unprecedented high-pressure environments in health systems and high stress for health professionals. Issues such as patent exemptions, compulsory licenses, technology transfers, and vaccine hubs have become key points of discussion on the development and supply of vaccines [1]. The pandemic has affected the global economy, labor market, and other facets of individual and societal lives [2]. In the context of COVID-19, the imbalance between the need to ensure routine care despite limited medical resources and the need to combat infectious diseases have raised ethical and clinical management issues [3]. However, these issues are not limited to the current COVID-19 pandemic. When the pandemic is over, these global health issues will become more important; moreover, as more people participate in the global health network, there may be conflicts of interest among individuals [1]. The United Nations (UN) formulated 17 Sustainable Development Goals (SDGs) between 2016 and 2030. Among them, SDG 3 aims to ensure healthy lives and promote well-being for all age groups [4]. Subsequently, the International Council of Nurses (ICN) recommended that the nursing associations of all countries be familiar with the contents of the SDGs that included the most important common goals of the UN and ICN [5].

Currently, global health is an emerging field that reflects the health and well-being of the global population. Thus, it is important to prepare the next generation of nurses as global health advocates [6]. Nurses are in direct contact with patients and provide care to them on the front lines [7]. As a result, nurses take care of inpatients and deal directly with the disease-prone population and their complications or mortality [8]. Nurses require cultural competency throughout this process. Cultural competency is the ability to understand the cultures of different minority groups and respond to their unique needs, establish and maintain relationships with people from different cultural backgrounds, avoid misunderstandings with people of other cultures while communicating efficiently, and to collaborate with individuals across cultures [9].

Improving cultural nursing competency can increase trust in the relationships developed between patients and nurses, facilitate health and disease observation and control, and increase the provision and safety of equal and appropriate health services [10]. Therefore, it is important that nurses who provide care to multicultural patients understand and respect diverse cultures and beliefs and have the competency to provide meaningful care to patients of different cultures through effective interactions [11].

However, in a study of nursing students in Korea, 51.8% of the students had no experience related to other cultures during their undergraduate years, and only 36.1% of the students had the rare opportunity to interact with other cultures within Korea [12]. When nurses encounter multicultural patients in clinical practice, they must provide their patients with optimal care based on their understanding of different cultures. Nevertheless, in general, the care that Korean nurses provide to patients of different cultures tends to be similar to the care they provide to Korean patients [13]. In contrast, patients from other countries and ethnicities have mentioned inequalities in delivery and access to healthcare services [14]. According to a study that analyzed education programs related to the internationalization of medical personnel, education programs in Korea lacked programs on internationalization competency that could strengthen the cultural awareness of medical and health personnel [15]. However, in other countries, when a global health competency program was continuously implemented, knowledge of and technical competency in global health were 66.76% and 61.4%, respectively [6], suggesting that continuous education was necessary. 

Therefore, it is necessary to continuously apply and develop programs to improve the global health and cultural competencies of nurses. To achieve this, global health, cultural competency, and their influencing factors in future medical professionals and nursing students must be assessed to provide basic data for developing adequate programs. Factors that affect global health and cultural competencies include metacognition, which is the ability to understand and manipulate one’s cognitive processes [16]; global health and cultural nursing confidence; and other aspects such as overseas volunteer experience, foreign language skills, and foreign friends that may induce familiarity with different cultures.

This study aimed to identify factors affecting the global health and cultural competencies of nursing students and to provide basic data for the development of programs to enhance these abilities.

## 2. Materials and Methods

### 2.1. Study Design

This was a cross-sectional descriptive study designed to identify the factors affecting the global health and cultural competencies of nursing students. 

### 2.2. Participants

A total of 108 fourth-year nursing students enrolled in the Department of Nursing at E University of S city in Gyeonggi-do and C University of C city in Chungcheonbuk-do were included in the study. First, the purpose of the study was explained to all students, and informed consent was obtained online before participation. The minimum number of participants was 102 when calculated using the G*power 3.1.9.4 program based on a study by Cho and Jang [17]; their study, which investigated the factors influencing cultural competency in nursing students, included a linear multiple regression analysis with an effect size of 0.24, significance of 0.05, power of 0.90, two-tailed test, and 12 random predictor variables (age, gender, English language ability, grade, foreign friends, foreign residence for more than one month, overseas volunteer experience, global health experience, global health confidence, cultural confidence, metacognition, and global health competency). In this study, an online questionnaire was administered, and data from 108 participants were analyzed.

### 2.3. Research Tools

#### 2.3.1. Global Health Competency

Global health competency was defined as understanding the burden of disease and the determinants of health and possessing core public health skills, including policy development, analysis, and program management and soft skills, including collaboration, partnering, communication, professionalism, capacity building, and political awareness [18]. In this study, global health competency was determined using a tool developed by Lee et al. [15], which was based on the global health core competency tool developed by Wilson et al. [19] and recognized by nursing professors in the USA. This study used the tool developed by Lee et al. [15], which was validated in Korean. The tool consisted of 24 items in six domains: three items on the burden of disease in the international community; six items on the effects of immigration, travel, and transportation on health; five items on health in society and environmental determinants; three items on health and the globalization of health care; five items on health care in environments with poor resources; and two items on human rights and health as a development resource. The items were evaluated on a four-point Likert scale from 1 for “strongly disagree” to 4 for “strongly agree”, with a higher score indicating greater global health competency. The Cronbach’s α value of the tool was 0.93 at the time of development by Lee et al. [15]. In the current study, Cronbach’s α values ranged from 0.36 to 0.81 for the six domains and was 0.90 for the overall tool.

#### 2.3.2. Cultural Competency

Cultural competency was defined as the ability to understand cultural influences and to provide care in harmony with a patient’s culture [20]. This study used items from the Caffrey Cultural Competence in Healthcare Scale developed by Caffrey et al. [21] and the Cultural Competence Assessment developed by Schim et al. [22], which evaluates cultural behavior and was assessed for reliability and validity by Park et al. [23]. The tool was used in this study with the permission of the original authors. The tool consisted of 42 four subdomains with 42 items: 12 items on cultural receptivity, 10 items on cultural knowledge, 6 items on cultural awareness, and 14 items on cultural behavior. The items were evaluated on a five-point Likert scale from 1 “strongly disagree” to 5 “strongly agree”, with higher scores indicating greater cultural competency. The Cronbach’s α value was 0.90 in the study by Park et al. [23]. In this study, the Cronbach’s α values ranged from 0.70 to 0.87 for the four domains and was 0.91 for the overall tool.

#### 2.3.3. Global Health Confidence

Global health confidence was defined as paying attention to the social determinants of health and included individuals, population groups, nursing practices, research, education, leadership, advocacy, and policy design. It referred to the confidence to perform nursing ethically by respecting human dignity, rights, and cultural diversity. In this study, global health confidence was evaluated using a 100 mm numeric scale line, from 0 points at the left end for “very unconfident” to 10 points at the right end for “very confident”. The item “What do you think about your current global health confidence?” was evaluated by marking a number on the scale from 0 to 10, with a higher score indicating greater global health confidence.

#### 2.3.4. Cultural Nursing Confidence

Cultural nursing confidence referred to the belief in performing appropriate and individualized nursing by each patient’s unique culture. In this study, cultural nursing confidence was evaluated using a 100 mm numeric scale line from 0 points at the left end for “very unconfident” to 10 points at the right end for “very confident”. The item “What do you think about your current cultural nursing confidence?” was evaluated by marking a number on the scale from 0 to 10, with a higher score indicating greater cultural nursing confidence.

#### 2.3.5. Metacognition

Metacognition was defined as the ability to understand and manipulate one’s cognitive processes and ways of thinking [16]. In this study, metacognition was evaluated using a tool developed by Schraw and Dennison [24] and translated into the Korean version by Jeong [25] for validation. The tool consisted of two subdomains with 52 items: 17 items on cognitive knowledge and 35 items on cognitive control. The items were evaluated on a six-point Likert scale ranging from 1 for “strongly disagree” to 6 for “strongly agree”, with higher scores indicating higher metacognition. The Cronbach’s α values for metacognitive knowledge and control in the study by Jeong [25] were 0.89 and 0.93, respectively. In this study, the Cronbach’s α values were 0.86 for metacognitive knowledge, 0.91 for metacognitive control, and 0.94 for the overall tool.

#### 2.3.6. Validity and Reliability of the Measurement Tools

Global health confidence and cultural nursing confidence, which consisted of one item each, were analyzed as the internal consistency of the two items, and the Cronbach’s α value was 0.87 (Table 1). The model fit index of the measurement model was confirmed by confirmatory factor analysis (CMIN (chi-squared test) = 92.82; df = 64, probability level = 0.011 for the measurement model; and CMIN/DF = 1.45, which satisfied less than 2). In addition, the model fit index was acceptable (goodness of fit index (GFI) 0.91, comparative fit index (CFI) 0.96, standard root mean residual (SRMR) 0.05, and root mean square error of approximation (RMSEA) 0.065. The average variance extracted (AVE) and composite reliability (CR) were calculated for the convergent validity of the measurement tools, and the AVE of each tool was from 0.50 to 0.86, which was above the standard 0.5, and the CR of each tool was from 0.80 to 0.93, which was above the standard 0.7. To check discriminant validity, it was checked whether AVE was greater than the square of the correlation coefficient. The square of the correlation coefficient of each tool was from 0.02 to 0.59, which was smaller than the AVE value of each tool, so discriminant validity was secured (Table 1).

### 2.4. Data Collection

Participant recruitment was announced on an online bulletin board for fourth-year students from 30 October to 7 November 2018. The researcher contacted fourth-year school representatives to check the curriculum and inform potential participants about the study’s background and purpose, methods, participation period, dropout policy, the anonymity of personal information, and confidentiality during break time. All questionnaires were explained as mandatory, and those who wished to participate voluntarily filled out the online questionnaire. Then, information on the questionnaire and participation method was announced on the online bulletin board once more. Although the participants included vulnerable groups, the researcher fully explained that the students would not incur any disadvantage from not participating or dropping out of the study and that participation in the study was not related to their grade evaluation. Each participant checked the online consent form with “I agree”. In the questionnaire, items related to personal information that could be used to identify the participants were minimized, and the questionnaire was submitted anonymously. When the online survey was completed, there were no missing values in the responses. Data from 108 participants were collected online and downloaded to a storage device; these were stored in a locker in the researcher’s laboratory to protect participants’ information.

### 2.5. Data Analysis

The collected data were analyzed using IBM SPSS (Armonk, NY, USA) Statistics 25.0. Participant characteristics and global health and cultural competencies, which were the outcome variables of this study, were assessed using frequency and percentage, mean and standard deviation, and minimum and maximum values. The Kolmogorov-Smirnov test was used to test for normality. An independent t-test was used to examine the global health and cultural competencies according to participant characteristics. The personal correlation coefficient was assessed to evaluate the correlation between global health competency, cultural competency, global health confidence, cultural nursing confidence, and metacognition. A hierarchical stepwise method was used to identify factors affecting global health and cultural competencies. A *p*-value <0.05 was considered to be statistically significant.

## 3. Results

### 3.1. Participant Characteristics

The mean age of the participants was 23.61 ± 1.50 (range of 21~33) years, with 69 participants (63.9%) under the mean age. Among the 104 participants, 94 (87.05) were women. The participants’ mean English proficiency score was 5.27 ± 1.53 (range of 2~8) points, with 59 students (54.6%) scoring higher than the mean. The mean grade for the previous semester was 3.56 ± 0.42 (range of 2.50~4.40), and 62 students (57.4%) scored above the mean. A total of 31 (29.7%) participants had friends from foreign countries, and 32 (29.6%) participants had previously stayed in foreign countries for more than a month. Nine (8.3%) participants had overseas volunteer experience, while five (4.6%) participants had experience with global health activities. The mean scores for global health confidence, cultural nursing confidence, and metacognition were 5.31 ± 1.94 (range of 1–10), 5.62 ± 2.02 (range of 1~10), and 219.98 ± 25.97 (range of 146~292), respectively (Table 2).

### 3.2. Global Health Competency and Cultural Competency

The total mean score for global health competency was 63.01 ± 8.78 (range of 24~87) points. When the scale was standardized, scores for social and environmental determinants of health and health implications from migration, travel, and displacement among the subdomains were 2.89 and 2.74 (range of 1~4) points, respectively. The global burden of disease was the lowest at 2.18 points. The total mean score for cultural nursing competency was 134.94 ± 17.78 (range of 79~191). When the scale was standardized, cultural nursing behavior had the highest score among the subdomains, at 3.49 (range of 1~5) points, while cultural knowledge had the lowest score at 2.97 points (Table 3).

### 3.3. Global Health and Cultural Competencies According to Participant Characteristics

Global health competency was higher in female than in male nursing students (t = −2.20, *p* = 0.030), and participants with higher-than-average learning strategies had higher global health competency than those with lower-than-average learning strategies (t = −2.34, *p* = 0.021). Cultural competency was significantly higher in participants with higher-than-average global health confidence (t = −3.04, *p* = 0.003), cultural nursing confidence (t = −4.13, *p* < 0.001), and metacognition scores (t = −4.12, *p* < 0.001) (Table 4).

### 3.4. Relationshisp among Global Health Competency, Cultural Competency, Global Health Confidence, Cultural Nursing Confidence, and Metacognition

Global health competency had a moderate positive correlation with cultural competency (r = 0.49, *p* < 0.001) and a weak positive correlation with cultural nursing confidence (r = 0.26, *p* = 0.006) and metacognition (r = 0.22, *p* = 0.023). Cultural competency showed a moderate positive correlation with global health confidence (r = 0.31, *p* = 0.001), cultural nursing confidence (r = 0.51, *p<* 0.001), and metacognition (r = 0.40, *p <* 0.001). Global health confidence had a strong positive correlation with cultural nursing confidence (r = 0.77, *p <* 0.001) and a moderate positive correlation with metacognition (r = 0.32, *p* *=* 0.001) (Table 5).

### 3.5. Factors Affecting Global Health Competency and Cultural Competency

To identify factors affecting the global health competency of nursing students, qualitative variables, such as gender, foreign friends, foreign residence for more than one month, overseas volunteer experience, and global health experience, were treated as dummy variables in Model 1. Age, English language ability, and grade were continuous variables; thus, a total of eight factors were included. In Model 2, cultural confidence, metacognition, and cultural competency were treated as continuous variables and included hierarchically to perform multiple regression analysis and construct a regression model for global health competency. A stepwise variable selection method was used to construct the model, and variables were selected based on a significant probability of 0.05 and removed based on a significant probability of 0.10. In regression models 1 and 2 for the global health competency of nursing students, tolerance was 0.94 and 1.00, respectively, which was greater than 0.01 in both models. The variance inflation factor (VIF) was 1.00 in Model 1 and 1.07 in Model 2, satisfying the assumption of regression analysis with statistical significance and without the problem of multicollinearity. In Model 1, age was a significant factor affecting global health competency (F = 5.19, *p* = 0.025). In Model 2, cultural competency was a significant influencing factor (F = 17.10, *p* < 0.001). The regression model with only cultural nursing confidence as the variable after controlling for age had an explanatory power of 23.1% for global health competency. 

A hierarchical model was constructed to identify the factors affecting the cultural competency of nursing students. Regression Models 1 and 2 for cultural competency had tolerances of 0.94 and 0.86–0.91, respectively. VIF was 1.06 in Model 1 and 1.09–1.17 in Model 2, that is, less than 10 in both models. Therefore, the assumption of regression analysis was satisfied with statistical significance, and there was no problem of multicollinearity. In Model 1, age and foreign residence for more than one month were factors with significant effects on cultural competency (F = 5.98, *p* = 0.003). In Model 2, cultural nursing confidence and metacognition were significant influencing factors (F = 14.97, *p* < 0.001). The regression model with cultural nursing confidence and metacognition as variables after controlling for age and foreign residence had an explanatory power of 34.3% for cultural competency (Table 6). 

## 4. Discussion

This study evaluated the global health and cultural competencies of nursing students and identified the influencing factors. First, global health competency had a mean score of 2.63 points (1–4 points), while cultural competency had a mean score of 3.21 points (1–5 points), which was slightly higher than the mean. This finding was similar to that of a previous study on the self-assessment of global health competency. In that study, knowledge and skill competencies for global health were 66.76% and 61.4%, respectively [6]. Since the beginning of the COVID-19 pandemic, the importance of understanding the world, global health, and interest in health inequality has been emphasized. The lack of information on viruses and diseases, uncertainty in treatment, and lack of vaccines are examples of these effects [2]. Therefore, to develop educational programs and interventions for the improvement of global health and cultural competencies, influencing factors were investigated.

First, cultural competency significantly affected global health competency, with an explanatory power of 23.1%. Global health competency encompasses understanding the burden of disease and health determinants, in addition to having core public health skills and soft skills [3]. It comprehensively covers various aspects of global health. However, cultural competency, which is the ability to provide care in harmony with a patient’s culture [20], was the only factor influencing global health competency. This suggests that the capacity to provide culturally sensitive care is the basis for global health competency. In previous studies, three areas of educational requirements were related to global health competency: (1) the relationship between health and access to clean water, sanitation, and nutrition; (2) cultural competency; and (3) understanding the relationship between health and human rights [6]. Our results align with the literature on the relationship between global health and cultural competencies [6] and further demonstrate that cultural competency is a significant factor affecting global health competency.

In a conceptual analysis, cultural competency is analyzed as the combination of cultural awareness, knowledge, sensitivity, skill, proficiency, and dynamicity [26]. This analysis implies that being aware of cultural competency and accepting and respecting the culture, health demands, culturally specific understandings of disease and health, and cultural differences to prevent other environmental factors from having significant effects on participants are the basis of global health competency [27]. 

Cultural competency—the ability to understand the characteristics of other cultures to not indiscriminately apply individual values or judgments, and to interact appropriately and effectively with people of other cultures [28]—plays a role in improving health competency; this suggests that specific knowledge, education, and having a basic understanding of and accepting other cultures, are all important factors.

In a previous study evaluating the effects of overseas programs on nursing students [12], understanding global health was the competency that most participants expected to learn during the program. However, the greatest achievement after participation was the expansion of human relationships. This indicates that global health is promoted not only by overseas programs but also by appropriate activities and programs that can improve global health competency.

Cultural nursing confidence and metacognition were factors affecting the cultural competency of nursing students, with an explanatory power of 34.3%. This finding aligns with that of a previous study [29] in which cultural nursing self-efficacy was found to be an important factor for cultural competency. Cultural nursing self-efficacy was a mediating factor that allowed cultural competency development to continue despite obstacles or stress [29]; thus, cultural nursing self-efficacy is also essential to improve cultural nursing confidence. Similarly, a study on cultural competency suggested a need for interventions that acknowledge the value of cultural awareness-based approaches, while also exploring the utility of more comprehensive cultural competence and safety approaches [30]. Altogether, these findings show that factors related to cultural competencies are complex. Short-term general culture-related education did not enhance cultural nursing self-efficacy. In contrast, to improve cultural nursing confidence, long-term systematic education that connects culture and nursing is necessary [31].

In this study, metacognition was a factor influencing cultural competency. Metacognition is the capacity to analyze one’s thoughts [16], to know and control one’s thinking processes, and to apply previously acquired knowledge, skills, and experience through appropriate strategies [32]. In particular, it is an important variable in learning and problem solving. It selects an appropriate strategy for the task; establishes, selects, and applies related knowledge and problem-solving strategies; evaluates the effects of the applied strategy; and regulates the performance process [33]. As cultural competency is the ability to understand the effects of culture and to provide care according to a patient’s culture [19], metacognition—which acts as a central factor in understanding, self-learning, communication, and problem-solving processes—may also be applied to improve cultural competency. Therefore, it is important to include elements that can improve metacognition in programs aimed at enhancing cultural competency.

In our study, the factors, age, gender, English language ability, grades, foreign friends, foreign residence for more than one month, overseas volunteer experience, and international health experience, were not factors affecting global health and cultural competencies. This finding suggests that experiencing foreign cultures and meeting others from different cultures are insufficient for promoting global health and cultural competencies. This conclusion aligns with that of previous research, indicating that short-term immersion and volunteer experience in foreign countries led to individuals’ growth while their understanding of the culture did not change [34]. As suggested in a study of internationalization programs for nursing students [12], curriculum internationalization that provided opportunities to meet nursing students from different cultures could play an important role in filling the gap between theory and practice. However, systematic programs with group activities based on such opportunities and writing a reflection journal are needed to improve related competencies. Feedback from students on international programs includes the importance of improving foreign language skills [12]. However, foreign language ability does not affect global health and cultural competencies. This means that foreign language ability or foreign experiences may help to improve global health and cultural competence, but they do not directly affect these competencies.

It is important not only to provide information on foreign cultures and increase awareness but also to educate students on how to accept differences using cultural competency. In one study, global health education improved global health competency in nursing students [35], indicating that global health competency can be improved through well-structured education. Experience of foreign cultures and the level of foreign language ability do not increase global competency, but these factors may create opportunities or means for communication. This reflects an intersection of metacognition and cultural nursing confidence that understands and interprets a situation comprehensively, allowing individuals to acknowledge and accept cultural differences.

In a modern globalized society, increased access to information does not improve global health competency. In contrast, specific education on interpreting global health issues, accepting these issues like one’s own and comprehensively analyzing these issues is necessary. Based on the findings of this study, it is necessary to apply an intervention program that can improve cultural competency through the enhancement of cultural nursing confidence and metacognition, subsequently improving global health competency.

The limitations of this study and suggestions for future research are as follows: First, several variables, other than the ones mentioned in this study, should be considered as factors affecting global health competency. Second, this was a cross-sectional study; thus, causal relationships couldnot be determined. Lastly, since this study had a small sample size as compared with other global health-related studies, care must be taken not to overestimate when interpreting the results. This study should be replicated by including different variables that align with the recent changes in the world, in terms of influencing factors. In the future, since cultural nursing confidence can be measured as a subdomain of cultural nursing competency, it is recommended to use the cultural nursing competency tool that includes cultural nursing confidence rather than the general cultural competency tool targeting nursing students.

## 5. Conclusions

We investigated the global health and cultural competencies of nursing students and identified the factors influencing these competencies about the rapidly changing global health environment and increasingly complex health issues. The participants showed moderate levels of global health and cultural competencies, and cultural competency was a significant factor affecting global health competency. Cultural nursing confidence and metacognition were significant factors affecting cultural nursing competency. While most previous studies have independently assessed global health and cultural competencies, we examined the two competencies simultaneously, demonstrating that cultural competency affects global health competency. This study also highlights the relevance of metacognition, which is a new concept that has not been previously addressed about global health and cultural competencies. The results of this study may be used for educational purposes in the future. Knowledge about global health and different cultures is important; however, it is also essential to improve metacognition, which provides nurses with confidence and allows them to make comprehensive judgments and to address the situation holistically.

## Figures and Tables

**Table 1:** Reliability, and validity of the variables.

Variable	Items	Cronbach’s α	CR	AVE
Global health competencies	24	0.90	0.86	0.50
Global burden of disease	3	0.61		
Health implications of migration, travel, and displacement	6	0.72		
Social and environmental determinants of health	5	0.81		
Globalization of health and health care	3	0.50		
Health care in low-resource settings	3	0.76		
Health as a human right and development resource	4	0.36		
Cultural competency	42	0.91	0.80	0.50
Cultural acceptance	12	0.77		
Cultural knowledge	10	0.77		
Cultural awareness	6	0.70		
Cultural nursing behavior	14	0.81		
Confidence	2	0.87	0.91	0.84
Global health confidence	1			
Cultural nursing confidence	1			
Meta-cognition	52	0.94	0.93	0.86
Metacognitive knowledge	17	0.86		
Metacognitive control	35	0.91		

Note: CR, composite reliability; AVE, average variance extracted.

**Table 2:** Characteristics of the participants.

Characteristics		N	%	M (SD)	Min~Max
Age (year)	<23.61	69	63.9	23.61 (1.50)	21~33
	≥23.61	39	36.1		
Gender	Male	14	13.0		
	Female	94	87.0		
English language ability	<5.27	59	54.6	5.27 (1.53)	2~8
	≥5.27	49	45.4		
Grade	<3.56	46	42.6	3.56 (0.42)	2.50~4.40
	≥3.56	62	57.4		
Foreign friends	Yes	31	28.7		
	Yes	32	29.6		
Foreign residence for more than one month	Yes	9	8.3		
International health experience	Yes	5	4.6		
Global health confidence	<5.31	54	50.0	5.31 (1.94)	1~10
	≥5.31	54	50.0		
Cultural nursing confidence	<5.62	49	45.4	5.62 (2.02)	1~10
	≥5.62	59	54.6		
Meta-cognition	<219.98	53	49.1	219.98 (25.97)	146~292
	≥219.98	55	50.9		

Note: N, number; M, mean; SD, standard deviation; Min~Max, minimum~maximum.

**Table 3:** Descriptive statistics of the variables.

Variable	Items	Mean	Standard Deviation	Min~Max	Scale Standardization
Global health competencies	24	63.01	8.78	24~87	2.63
Global burden of disease	3	6.55	1.54	3~10	2.18
Health implications of migration, travel, and displacement	6	16.43	2.55	6~24	2.74
Social and environmental determinants of health	5	14.46	2.33	5~20	2.89
Globalization of health and health care	3	7.48	1.48	3~12	2.49
Health care in low-resource settings	3	7.68	1.57	3~12	2.56
Health as a human right and development resource	4	10.42	1.82	4~15	2.60
Cultural competency	42	134.94	17.78	79~191	3.21
Cultural acceptance	12	37.65	6.60	15~55	3.14
Cultural knowledge	10	29.74	5.91	14~46	2.97
Cultural awareness	6	18.68	2.95	9~27	3.11
Cultural nursing behavior	14	48.88	6.53	27~65	3.49
Confidence	2	10.93	3.73	0~20	
Global health confidence	1	5.31	1.94	0~10	
Cultural nursing confidence	1	5.62	2.02	0~10	
Meta-cognition	52	134.94	17.78	79~191	4.23
Metacognitive knowledge	17	76.13	9.33	55~100	4.48
Metacognitive control	35	152.67	18.55	98~204	4.36

Note: Min~Max, minimum~maximum.

**Table 4:** Differences in outcome variables by participant characteristics.

Characteristics		N	Global Health Competencies			Cultural Competencies		
			M (SD)	t	*p*	M (SD)	t	*p*
Age (year)	<23.61	69	63.26 (8.35)	0.39	0.694	135.99 (16.83)	0.81	0.421
	≥23.61	39	62.56 (9.59)			133.10 (19.44)		
Gender	Male	14	58.29 (8.71)	−2.20	0.030	126.86 (15.55)	−1.84	0.068
	Female	94	63.71 (8.61)			136.15 (17.85)		
English language ability	<5.27	59	63.88 (8.54)	1.13	0.259	134.10 (19.24)	−0.54	0.591
	≥5.27	49	61.96 (9.03)			135.96 (15.98)		
Grade	<3.56	46	62.13 (9.84)	−0.90	0.373	133.63 (20.22)	−0.66	0.511
	≥3.56	62	63.66 (7.92)			135.92 (15.83)		
Foreign friends	No	77	63.17 (8.01)	0.30	0.767	133.12 (16.80)	−1.70	0.092
	Yes	31	62.61 (10.58)			139.48 (19.57)		
Foreign residence for more than one month	No	76	62.70 (8.74)	−0.57	0.572	133.46 (16.70)	−1.34	0.183
	Yes	32	63.75 (8.96)			138.47 (19.97)		
Overseas service experience	No	99	63.05 (8.85)	0.16	0.872	134.89 (17.20)	−0.11	0.915
	Yes	9	62.56 (8.38)			135.56 (24.56)		
International health experience	No	103	62.99 (8.98)	−0.35	0.727	134.77 (18.16)	−0.47	0.640
	Yes	5	63.40 (1.67)			138.60 (5.32)		
Global health confidence	<5.31	54	61.52 (8.18)	−1.78	0.077	129.93 (19.79)	−3.04	0.003
	≥5.31	54	64.50 (9.17)			139.96 (13.97)		
Cultural nursing confidence	<5.62	49	62.04 (9.11)	−1.05	0.298	127.71 (19.28)	−4.13	<0.001
	≥5.62	59	63.81 (8.48)			140.95 (13.95)		
Metacognition	<220	53	61.66 (8.54)	−1.58	0.117	128.25 (18.82)	−4.12	<0.001
	≥220	55	64.31 (8.89)			141.40 (14.11)		

Note: N, number; M, mean; SD, standard deviation.

**Table 5:** Correlations between variables.

Variable	Global Health Competencies	Cultural Competencies	Global Health Confidence	Cultural Nursing Confidence	Metacognition
	*r* (*p*)				
Global health competencies	1				
Cultural competencies	0.49 (<0.001)	1			
Global health confidence	0.14 (0.141)	0.31 (0.001)	1		
Cultural nursing confidence	0.26 (0.006)	0.51 (<0.001)	0.77 (<0.001)	1	
Metacognition	0.22 (0.023)	0.40 (<0.001)	0.32 (0.001)	0.32 (0.001)	1

**Table 6:** Influencing factors on global health competencies and cultural competencies.

Variable	Global Health Competencies				Cultural Competencies			
	Model 1		Model 2		Model 1		Model 2	
	B (SE)	t (*p*)	B (SE)	t (*p*)	B (SE)	t (*p*)	B (SE)	t (*p*)
Intercept	92.92 (13.16)	7.06 (<0.001)	46.02 (14.76)	3.12 (0.002)	217.07 (26.52)	8.19 (<0.001)	134.31 (26.74)	5.02 (<0.001)
Age	−1.27 (0.56)	−2.28 (0.025)	−0.58 (0.51)	−1.13 (0.260)	−3.58 (1.13)	−3.16 (0.002)	−2.47 (0.97)	−2.54 (0.013)
Foreign residence for more than one month					7.78 (3.69)	2.11 (0.037)	2.34 (1.24)	0.72 (0.472)
Cultural competencies			0.23 (0.04)	5.26 (<0.001)				
Cultural confidence							3.50 (0.75)	4.68 (<0.001)
Metacognition							0.18 (0.06)	3.09 (0.003)
*F* (*p*)	5.19 (0.025)		17.10 (<0.001)		5.98 (0.003)		14.97 (<0.001)	
Adj. R^2^	0.038		0.231		0.085		0.343	
Tolerance	1.00		0.94		0.94		0.86~0.91	
VIF	1.00		1.07		1.06		1.09~1.17	
Durbin-Watson	2.052				2.277			

Note: B, unstandardized regression coefficient; SE, standard error; Adj.R^2^, adjusted R^2^; VIF, variance inflation factors.

## Data Availability

Data sharing is not applicable.
